# Transdifferentiation is a driving force of regeneration in *Halisarca dujardini* (Demospongiae, Porifera)

**DOI:** 10.7717/peerj.1211

**Published:** 2015-08-25

**Authors:** Ilya E. Borisenko, Maja Adamska, Daria B. Tokina, Alexander V. Ereskovsky

**Affiliations:** 1 Department of Embryology, Faculty of Biology, Saint-Petersburg State University, Saint-Petersburg, Russia; 2 Sars International Centre for Marine Molecular Biology, University of Bergen, Bergen, Norway; 3 Current affiliation: Research School of Biology, Australian National University, Canberra, Australia; 4 Current affiliation: Institut Méditerranéen de Biodiversité et d’Ecologie Marine et Continentale (IMBE), CNRS, Aix Marseille Université, IRD, Avignon Université, Marseille, France

**Keywords:** Sponges, Regeneration, Morphogenesis, Epithelial-to-mesenchymal transition, Transdifferentiation, *Halisarca dujardini*

## Abstract

The ability to regenerate is widespread in the animal kingdom, but the regenerative capacities and mechanisms vary widely. To understand the evolutionary history of the diverse regeneration mechanisms, the regeneration processes must be studied in early-evolved metazoans in addition to the traditional bilaterian and cnidarian models. For this purpose, we have combined several microscopy techniques to study mechanisms of regeneration in the demosponge *Halisarca dujardini*. The objectives of this work are to detect the cells and morphogenetic processes involved in *Halisarca* regeneration. We show that in *Halisarca* there are three main sources of the new exopinacoderm during regeneration: choanocytes, archaeocytes and (rarely) endopinacocytes. Here we show that epithelial-to-mesenchymal transition (EMT) and mesenchymal-to-epithelial transition (MET) occur during *Halisarca* regeneration. EMT is the principal mechanism during the first stages of regeneration, soon after the injury. Epithelial cells from damaged and adjacent intact choanocyte chambers and aquiferous canals assume mesenchymal phenotype and migrate into the mesohyl. Together with archaeocytes, these cells form an undifferentiated cell mass beneath of wound, which we refer to as a blastema. After the blastema is formed, MET becomes the principal mechanism of regeneration. Altogether, we demonstrate that regeneration in demosponges involves a variety of processes utilized during regeneration in other animals (e.g., cell migration, dedifferentiation, blastema formation) and points to the particular importance of transdifferentiation in this process. Further studies will be needed to uncover the molecular mechanisms governing regeneration in sponges.

## Introduction

Regeneration is a biological phenomenon which is critical for the survival of an organism. Reparative regeneration is widely distributed among metazoans, with both similar and divergent cellular and morphogenetic mechanisms utilized within and among different taxa (Sànchez Alvarado, 2000; Tanaka & Reddien, 2011; Vervoort, 2011). To understand the evolutionary history of the diverse regeneration mechanisms, the regeneration processes must be studied in early-evolved metazoans in addition to the traditional bilaterian and cnidarian models.

The regeneration of injured or lost body parts is particularly common in epifauna, and is essential for community recovery from external disturbances that damage these animals (Henry & Hart, 2005). Sponges (phylum Porifera) are benthic sedentary animals which are not equipped with protective tissues or structures like cuticles, scales or shells, and are covered only by a single-cell layer (exopinacoderm). It has been suggested that this lack of protection against injury closely correlates with the high regenerative capacity of sponges (Korotkova, 1997). However, the patterns and rate of regeneration are strikingly different among different sponge species (Henry & Hart, 2005; Wulff, 2013). The regenerative capacities of sponges range from an ability to restore a lost body part, to development from a piece of tissue, or restoration of a complete organism from an aggregate of dissociated cells (Korotkova, 1997).

A large number of studies on regeneration in Porifera, carried out in the last decade, were performed on somatic cell conglomerates called primmorphs (for review see: Nickel & Brümmer, 2003; Chernogor et al., 2011; Lavrov & Kosevich, 2014; Eerkes-Medrano, Feehan & Leys, 2015). Primmorphs represent a suitable model to study de-differentiation or differentiation of cells, as these events do take place during primmorph development. However, as an artificial system primmorphs are not an ideal model for the study of reparative regeneration in whole organisms or its parts. In spite of the importance of sponges as excellent models for investigation of the evolutionary history of regeneration mechanisms and morphogenesis, studies of regeneration after surgical injury or development of sponges from body fragments have been generally limited to histological descriptions using light microscopy (Korotkova, 1961; Korotkova, 1963; Korotkova, 1970; Korotkova, 1972; Korotkova & Nikitin, 1969a; Korotkova & Nikitin, 1969b; Diaz, 1979). There are only a few ultrastructural studies, describing regeneration of sponge structures such as oscular diaphragm (Thiney, 1972) or inhalant papillae (Boury-Esnault, 1976). It was shown that in many sponges, regeneration and primmorph formation from dissociated cells is accompanied by cell transdifferentiation (reviewed in: Simpson, 1984; Korotkova, 1997). Transdifferentiation is the conversion of one type of already differentiated cell to another type of normal differentiated cell. In some cases, transdifferentiation is accompanied by cell division, whereas in others it is not (Tosh & Slack, 2002; Shen, Burke & Tosh, 2004).

*Halisarca dujardini* Johnston, 1842 (class Demospongiae) is a convenient sponge for studying regeneration. It is a common species in littoral habitats along the European coasts from the English Channel to the White Sea (Ereskovsky et al., 2011), and is accessible throughout the year. Absence of spicules and skeleton makes this sponge useful for histological processing. Embryonic development, metamorphosis and morphology of *H. dujardini* have been described in detail (Lévi, 1956; Ereskovsky & Gonobobleva, 2000; Ereskovsky, 2002; Gonobobleva & Ereskovsky, 2004a; Gonobobleva & Ereskovsky, 2004b; Mukhina et al., 2006; Gonobobleva & Maldonado, 2009; Ereskovsky, Konjukov & Willenz, 2007; Ereskovsky et al., 2011).

It has been previously demonstrated that *H. dujardini* has high regenerative capacity. A study on the cellular response to the introduction of foreign material into the sponge body has shown that the sponge response combines protective and regenerative processes (Korotkova & Movchan, 1973). Transdifferentiation of choanocytes into exopinacocytes and endopinacocytes has been shown to occur during regeneration of the sponge from conglomerates of dissociated cells (Volkova & Zolotoreva, 1981). Finally, regeneration of *H. dujardini* from small body fragments has been described at the light microscopy level (Korotkova, Suchodolskaya & Krasukevitch, 1983; Sukhodolskaya & Krasyukevich, 1984). This type of injury leads to the restructuring of most of the aquiferous system. Labeling choanocytes by China ink suspension, the authors demonstrated a variety of fates of choanocytes: a significant proportion remains in the mesohyl as dedifferentiated cells, others are included in the exopinacoderm, and a few are transdifferentiated into endopinacocytes of the aquiferous canals. Epithelialization of the wound surfaces was suggested to be due to the stretching of the adjacent exopinacoderm, and also differentiation of archaeocytes (Korotkova, Suchodolskaya & Krasukevitch, 1983; Sukhodolskaya & Krasyukevich, 1984).

In the current work, we revisited the regeneration of *Halisarca dujardini* using transmission and scanning electron microscopy, allowing us to more precisely address the issues of cell transdifferentiation and movement. In addition, we have studied cell proliferation during regeneration in this species. Our study demonstrates a variety of morphogenetic processes during reparative regeneration, and identifies cells involved in these processes.

## Material and Methods

### Sponge materials

Sponges *Halisarca dujardini* Johnston (Demospongiae, Chondrosida) were collected in the White Sea (Chupa Inlet, Kandalaksha Bay) in June–July 2010–2012 and in April of 2010 and March of 2011 near island Sotra in the North Sea (Bergen, Norway). Sponges were collected with their algal substrate and maintained in aquariums with sea water at +12 °C in the Marine Station of the Zoological Institute RAS (Kartesh, White Sea) and in the Sars International Center for Marine Molecular Biology (Bergen, Norway).

### Field study permissions

No specific permissions were required for these locations because the study was done outside of the national park, private land or protected area. We confirm that the field studies did not involve endangered or protected species.

### Surgical procedures

Manual dissections were performed with the aid of a stereomicroscope and use of Castroviejo scissors and microscalpels. For each experiment, a portion of apical ectosome (superficial part of the sponge) was removed, along with a directly underlying section of the aquiferous system (choanocyte chambers and canals). The depth of excision varied slightly between the operations, from 150 to 500 µm. The osculum (exhalant opening) remained intact in all cases. Wounded sponges were maintained in 40 mm Petri dishes with 0.22 µm-filtered sea water replaced daily. Six individuals were observed for each time point at various intervals until regeneration was complete. Timing was started from 0 h (wounding); regeneration was monitored under a dissecting microscope and specimens were fixed at 3, 6, 12, 24, 48 and 72 h after excision.

### Light and electron microscopy

For microscopic investigation sponges were fixed in a solution composed of one volume of 25% glutaraldehyde, four volumes of 0.2 M cacodylate buffer and five volumes of filtered seawater for 2 h and post-fixed in 2% OsO_4_ in seawater at room temperature for 2 h. After fixation, samples were washed in 0.2 M cacodylate buffer and distilled water successively, and finally dehydrated through a graded ethanol series. For semi thin sections and transmission electron microscopy (TEM) specimens were embedded in Araldite resin. Semi thin sections (1 µm in thickness) were cut on a Reichert Jung ultramicrotome equipped with a diamond knife “Micro Star” 45°, then stained with toluidine blue, and observed in a WILD M20 microscope. The digital photos were made on a Leica microscope DMLB with the system of photo capture Evolution LC color. Ultrathin sections (60–80 nm) were cut with a Leica UCT ultramicrotome equipped with a diamond knife Drukkert 45°. Ultrathin sections, contrasted with uranyl acetate, were observed under a Zeiss-1000 transmission electron microscope (TEM). For scanning electron microscopy (SEM), fixed specimens were critical-point-dried, sputter-coated with gold-palladium, and observed under a Hitachi S 570 SEM.

### Cell proliferation investigation

For labeling of newly synthesized DNA we used 5-Ethynyl-2′-deoxyuridine (EdU; Molecular Probes), which is incorporated into genomic DNA during S-phase (Salic & Mitchison, 2008). A series of experiments with different concentrations of EdU and incubation times was performed to establish optimal conditions of labeling. In most cases, incubation time between addition of EdU and fixation was 6 h although images with longer incubation are also presented. EdU was added 6 h before the end of the experiment; thus, in the case of a 6 h of regeneration, EdU was added immediately after surgery, and in experiments with 12 h of regeneration—6 h after surgery and 6 h before fixation. This experimental setup resulted in labeling of DNA synthesized during the last 6 h of regeneration in each experiment. Stock solution of EdU in DMSO (500 mM) was added to filtered sea water with sponges to a final concentration of 800 µM. Six sponges were used for each stage of regeneration. Non-operated sponges, placed at the same time in the seawater with EdU as the operated experimental sponges, served as experimental controls. Sponges cultivated in sea water without EdU served as negative technical controls.

Specimens at different stages of regeneration and immediately after wounding were fixed in 4% paraformaldehyde in PBS (phosphate buffered saline with pH 7.4) for 1 h followed by washing in PBS. Fixed and rinsed specimens were blocked in 5% normal sheep serum/0.05% Tween-20 in PBS during 1 h at room temperature. Specimens were treated with the Click-iT^®^ EdU Alexa Fluor^®^ 488 Imaging Kit (Molecular Probes, Carlsbad, California, USA) according to manufacturer instructions, and then incubated in mouse anti-tubulin antibody (Sigma-Aldrich, Seelze, Germany) at 4 °C overnight. Specimens were then rinsed three times in blocking buffer during 3–5 h at while rotating and incubated in Alexa Fluor^®^ labeled anti-mouse secondary antibody (Molecular Probes, Carlsbad, California, USA). After 12 h of incubation in the secondary antibody, specimens were rinsed and the DNA was stained with TO-PRO^®^ -3 (Molecular Probes, Carlsbad, California, USA) at concentration 1 µM in PBS during 2 h. Rinsed samples were mounted in DABCO-glycerol and images obtained using a multiphoton confocal microscope Leica TCS SP5 MP with white laser WLL. Pictures processed with LAS AF Lite (Leica Microsystems, Wetzlar, Germany) and ImageJ software (http://imagej.nih.gov/ij/).

## Results

### Anatomy and cytology of *Halisarca dujardini*

**Figure 1:**
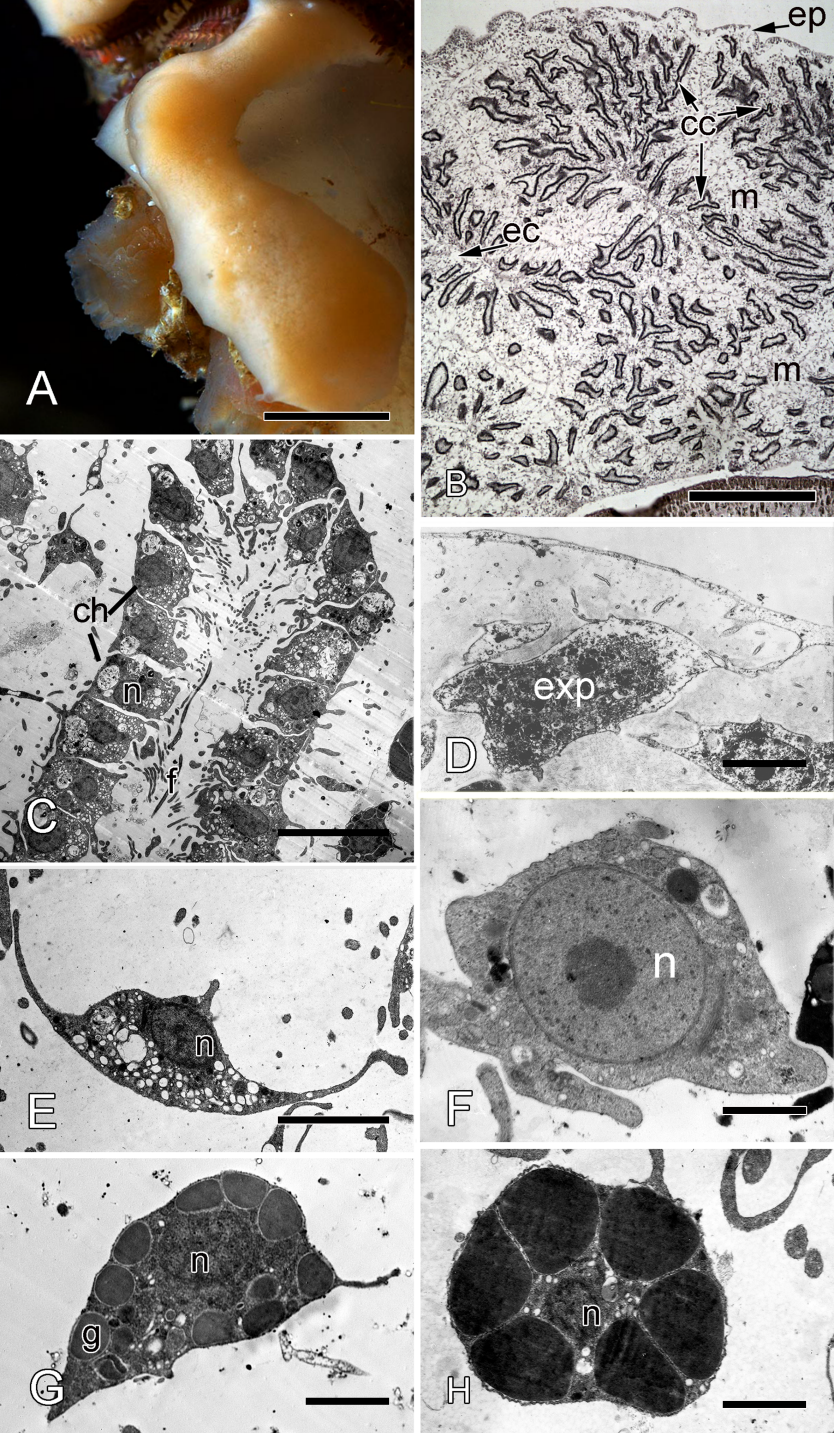
*Halisarca dujardini* habitus and the morphology of intact sponge. (A) *Halisarca dujardini in vivo* and *in situ*. (B) Histological section of sponge. (C) TEM of a choanocyte chamber. (D) TEM of an exopinacocyte; (E) TEM of an endopinacocyte; (F) TEM of an archaeocyte; (G) TEM of a granular cell; (H) TEM of a spherulous cell. Scale bars: A–10 mm; B–5 mm; C—10 µm; D—2 µm; E—4 µm; F—1 µm; G, H—2 µm. cc, choanocyte chamber; ch, choanocytes; ec, exhalant canal; enp, endopinacocyte; ep, exopinacoderm; exp, exopinacocyte; f, flagella; g, granules; m, mesohyl; n, nucleus.

**Figure 2:**
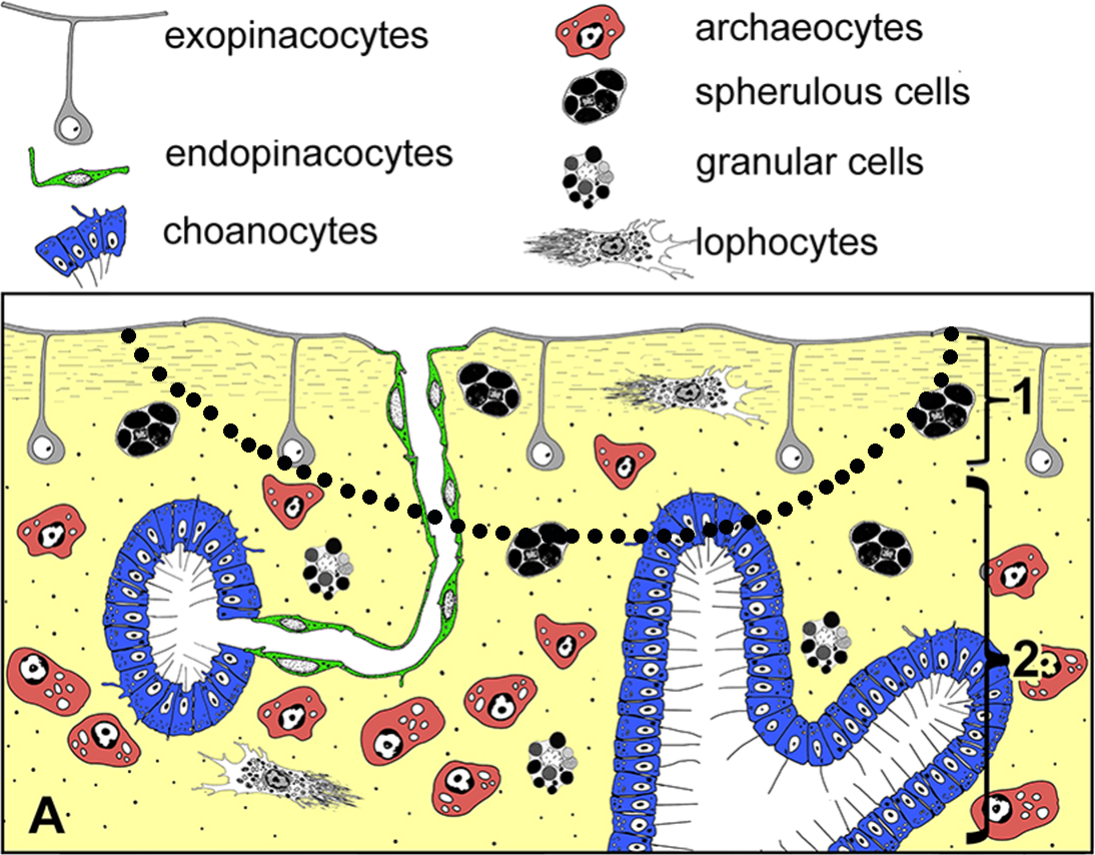
Simplified diagrams of histological organization of *Halisarca dujardini*: sponge structure and cell composition before injury (A) Dotted line shows the area of excision. 1—ectosome; 2—choanosome.

*Halisarca dujardini* is a predominantly shallow water, encrusting sponge growing up to 0.5–4 cm in diameter with thickness of 0.5–12 mm (Fig. 1A). Inorganic and organic skeletons are absent, and the surface is smooth. The body is composed of the peripheral ectosome and the internal choanosome (Figs. 1B and 1C; see also cartoon representation on Fig. 2). The ectosomal region is up to 27 µm thick and consists of three layers: (1) external parts of T-shaped exopinacocytes (Fig. 1D) which are covered an acellular mucous cuticle; (2) layer containing collagen fibrils and rare spherulous cells; and (3) the inner layer, consisting of condensed collagen fibrils and the cell bodies of exopinacocytes. The choanosome (Fig. 1B) makes up the greatest volume of the sponge body and is composed of choanocyte chambers (built of choanocytes the only cell type which is flagellated, Fig. 1C), aquiferous canals (built of endopinacocytes, Fig. 1E) and the mesohyl. Populations of free cells in the mesohyl of *H. dujardini* include: archaeocytes, lophocytes, spherulous cells, granular cells, microgranular cells, and vacuolar cells (Ereskovsky et al., 2011) (Figs. 1E–1H). Neither specialized cell junctions, nor basement membranes could be identified in association with any of the cell types.

### Description of regeneration

The observed regeneration processes in *Halisarca dujardini* can be subdivided into three stages: I—formation of “regenerative plug” (0–12 h), II—wound healing and formation of a “blastema” (12–36 h), and III—restoration of ectosome and choanosome (36–72 h).

### Stage I—formation of a “regenerative plug”

**Figure 3:**
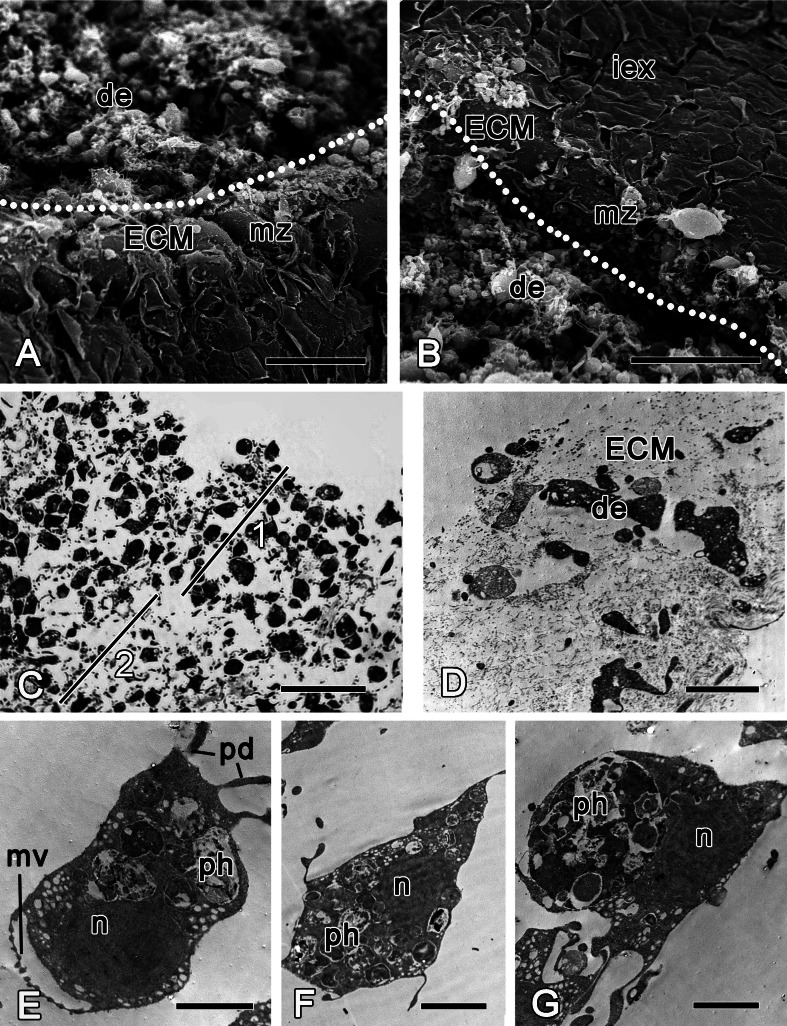
*Halisarca dujardini* 6 h after injury. (A) SEM of a wound surface (show with dotted line) with debris (de) and marginal zone, surrounding a wound with dense ECM of the cortex and the exopinacocytes. (B) SEM of intact exopinacoderm, marginal zone and the wound (show with dotted line), covered with debris. (C) Semi thin section of wounded ectosome (1) and adjacent area of choanosome (2). (D) TEM of external part of the wound with cell debris and dense ECM. (E) TEM of dedifferentiating choanocyte in the wound area. (F) TEM of amoebocyte of mesohyl, filled with phagosomes. (G) Endopinacocyte from the wound area filled with big phagosomes. Scale bars: A, B—30 µm; C—25 µm; D–G—2 µm. de, debris; ECM, extra cellular matrix; iex, intact exopinacoderm; mv, microvilli; mz, marginal zone; n, nucleus, pd, pseudopodia; ph, phagosome.

Sections of ectosome together with the directly adjacent choanosome were surgically removed at the beginning of the experiment (Fig. 2). Immediately after the injury, the wound surface retracts, leaving the surface of the intact ectosome protruding around the edges of the wound (Figs. 3A and 3B). The ectosome and the upper areas of choanosome are destroyed (Fig. 3C). However, the structure of the aquiferous system in the deeper zone of choanosome is preserved. The wound surface is covered with exudate and cell debris. A number of amoeboid cells can be identified among the extracellular matrix fibers (ECM) (Figs. 3A–3D). Choanocyte chambers and aquiferous canals in the damaged zone disintegrate: cells of these structures lose contacts with adjacent cells and change their shape from trapeziform (choanocytes) and flat (endopinacocytes) to spherical or amoeboid (Figs. 3E–3G). These changes mark the beginning of dedifferentiation (followed by transdifferentiation, see next section), which is accompanied by migration of the cells into the mesohyl. The de- and trans-differentiating cells can be tracked thanks to preservation of their characteristic organelles. The natural label of dedifferentiated choanocytes is the flagellar apparatus (basal body and accessory centriole situated next to the nucleus), which remains in the cell. The collar of microvilli is reduced and disappears, and the flagellum resorbs (Fig. 3E), although some dedifferentiated choanocytes keep their flagella up to the last stage of transdifferentiation into an exopinacocyte (see: Fig. 9H). Endopinacocytes, which are flat in their intact state, assume spherical to amoeboid shapes and can be identified by their small anucleolated nuclei.

During the initial six hours after injury, the “regenerative plug” forms at the wound area. It consists of exudate (mucus), bacteria and cell debris, and includes dedifferentiated pinacocytes, choanocytes, spherulous and granular cells of the mesohyl (Figs. 3A and 3D). In the wound area, vacuolar cells, phagocytes and archaeocytes can also be found. With exception of the spherulous cells, all cells are filled with phagosomes, testifying to their involvement in active phagocytosis of the cell debris (Figs. 3E–3G).

**Figure 4:**
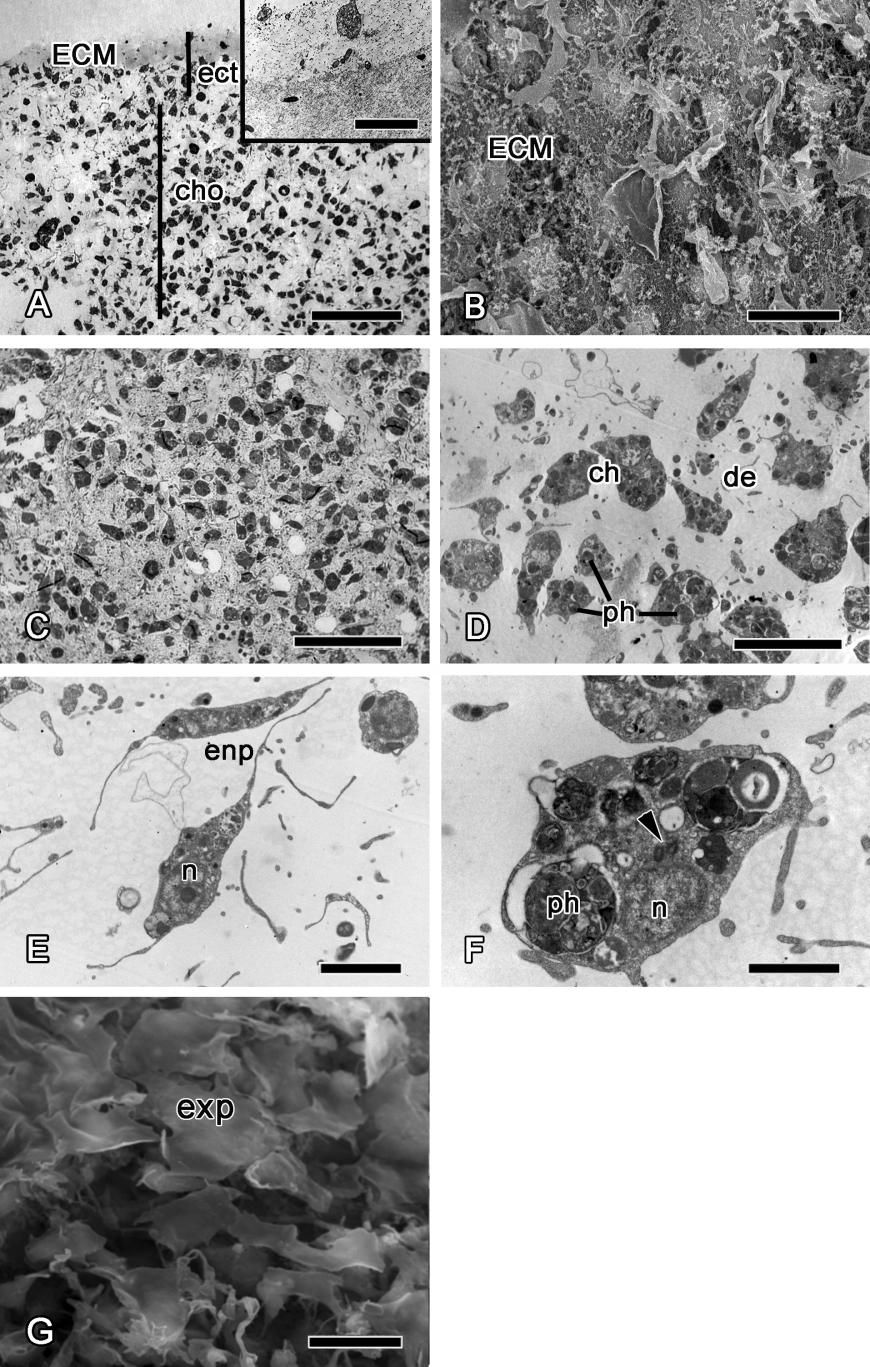
12 h after injury. (A) Semi thin section of wounded ectosome and adjacent area of choanosome. Inset: middle layer of ectosome, containing collagen fibrils organized into firm tracts. (B) SEM of wound surface, covered with ECM. (C) Semi thin section of wounded choanosome. (D) TEM of dedifferentiated cells in wounded choanosome. (E) TEM of dedifferentiated endopinacocytes in wounded choanosome. (F) TEM of dedifferentiated choanocyte filled with phagosomes, but with remaining basal body and accessory centriole (arrowhead). (G) SEM of intact exopinacocytes, surrounding the wound. ch, choanocytes; cho, choanosome; de, debris; ect, ectosome; ECM, extra cellular matrix; enp, endopinacocytes; exp, exopinacocytes; n, nucleus; ph, phagosomes. Scale bars: A—50 µm, B—10 µm; C—25 µm; D—15 µm; E—4 µm; F—2 µm; G—10 µm.

At 12 h after injury, the wound surface is completely covered with exudate and ECM (Figs. 4A and 4B), with minor amount of debris still detectable at the surface. Underlying choanosome areas are disorganized (Figs. 4C–4F). Intact exopinacocytes, surrounding the wound, change the shape of their flat cytoplasmic outgrowths, and the peripheral parts of adjacent cells become separated (Fig. 4G). Reorganization of the ectosome begins with restoration of its middle layer containing interlaced collagen fibrils organized into firm tracts (Fig. 4A).

### Stage II—wound healing and blastema formation

**Figure 5:**
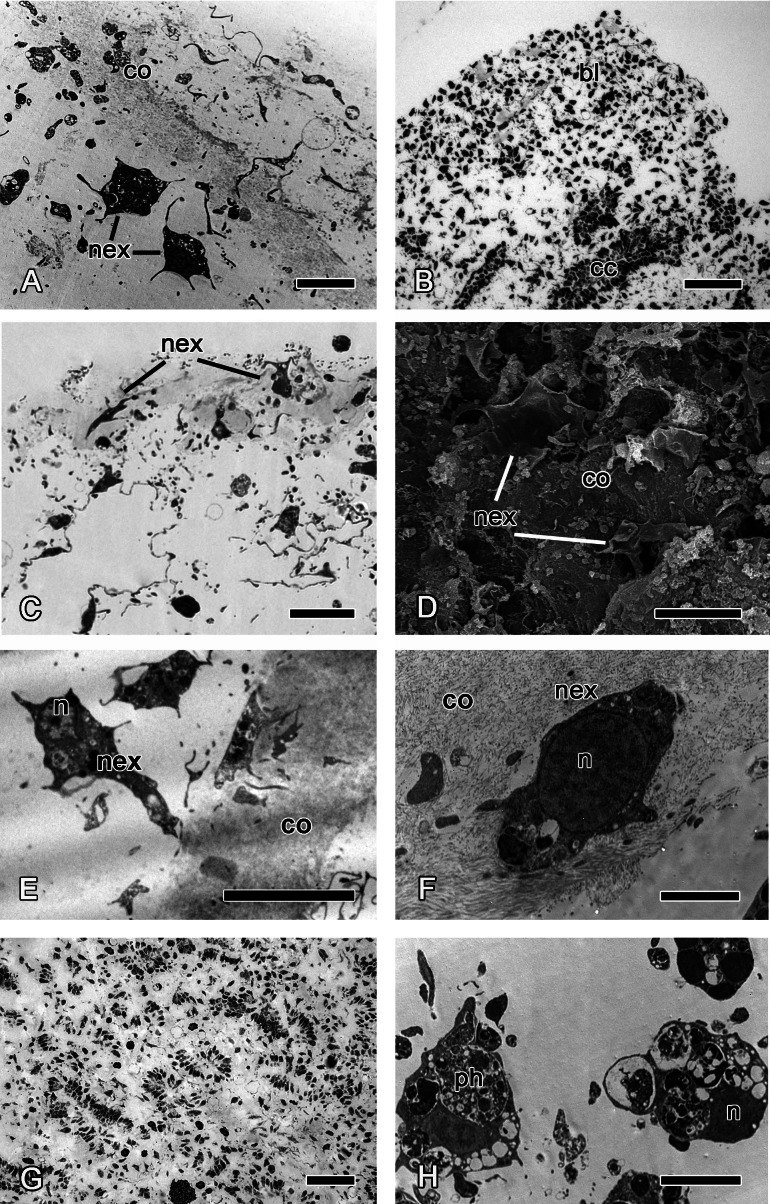
24 h after injury. (A) TEM of regenerating ectosome with newly differentiated exopinacocytes. (B) Semi thin section of regenerated ectosome and adjacent area of choanosome with concentration of dedifferentiated cells (blastema). (C) Semi thin section of regenerated ectosome with newly differentiated exopinacocytes. (D) SEM of the regenerated wound surface with newly differentiated exopinacocyte and dense collagen of the cortex (co); (E), (F) TEM of different modes of exopinacocytes differentiation from choanocyte (E) with character nucleus shape, and archaeocyte (F). (G) Semi thin sections of damaged choanosome without aquiferous system structures. (H) TEM of dedifferentiated cells of choanosome, filled with phagosomes. Scale bars: A—5 µm; B—50 µm; C—15 µm; D—15 µm; E—10 µm; F—2 µm; G—100 µm; H—5 µm. bl, blastema; cc, choanocyte chamber; co, collagen layer of ectosome; n, nucleus; nex, new exopinacocytes; ph, phagosome.

24 h after wounding, the ectosome is composed of a well-developed dense collagen layer, which is comparable to that of the intact ectosome (Fig. 5A compare with Figs. 8A and 8B).

Archaeocytes, which are toti- or pluripotent cells of demosponges (Funayama, 2013), and the dedifferentiated cells (choanocytes, pinacocytes) accumulate beneath the wound surface (Fig. 5B). This accumulation of cells strikingly resembles blastemas formed during regeneration of other animals. In line with this similarity, the new exopinacoderm (ectosome) and aquiferous system (choanosome) structures form from the cells of the blastema.

**Figure 6:**
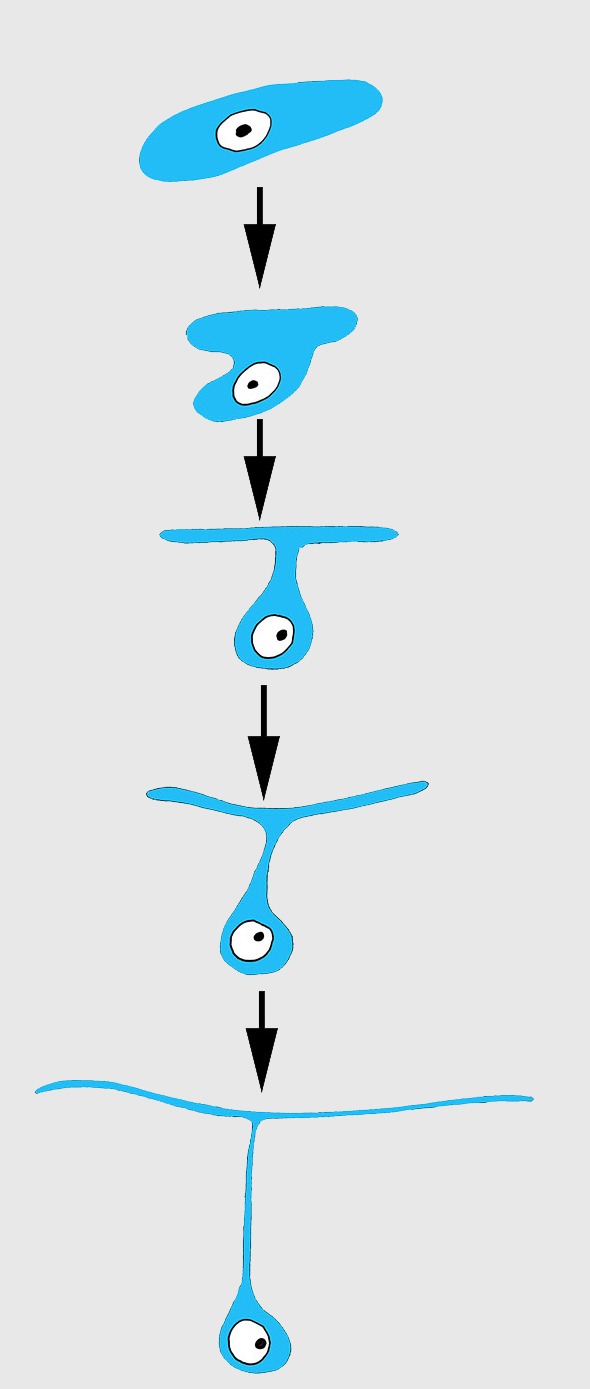
Scheme of principal mode of different cells transformation into T-shaped exopinacocytes by cell flattening parallel to sponge surface, immersion of main cytoplasmic volume of cell with the nucleus inside the ectosome, and formation of a fine cytoplasmic bridge between the apical plate and the immersed part of the cell.

Differentiation of exopinacocytes is individual, and begins with cells migrating from the blastema towards the wound surface. Upon reaching the wound, the cells flatten, assuming positions parallel to the surface (Figs. 5C and 5D). Ultrastructural characters demonstrate that the migrating cells include archaeocytes, dedifferentiated choanocytes (Figs. 5E and 5F), endopinacocytes and exopinacocytes and newer other mesohyl cells types. Then, these cells begin to transform into T-shaped exopinacocytes, characteristic of the adult sponge: most of the cytoplasm of the cell and the nucleus are inside the ectosome. A fine cytoplasmic bridge is retained between the apical plate and the immersed part of the cell (Fig. 6). The apical parts of new exopinacocyte extend into large polygonal plates.

At 24 h after injury, the part of choanosome which is underlying the wound remains anarchized, presenting as an accumulation of individual cells rather than continuous epithelium (Fig. 5G). These cells include many phagosomes (Fig. 5H); some cell debris remains visible in the area.

### Stage III—restoration of ectosome and choanosome

**Figure 7:**
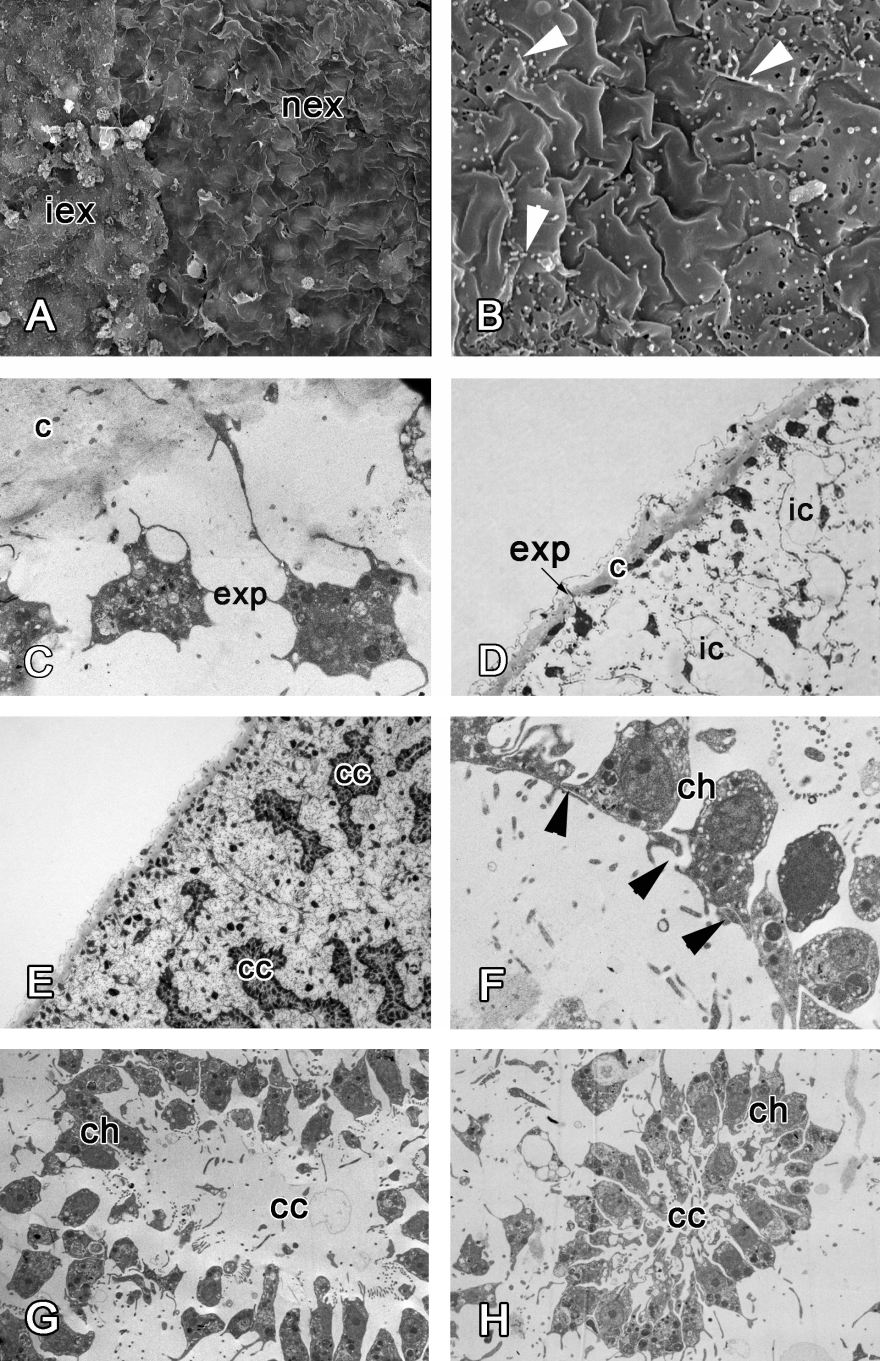
48 h after injury. (A) SEM of the intact and new exopinacoderm. (B) SEM of details of new exopinacoderm, showed numerous small cytoplasm microvilli form at the upper (apical) surface (arrowheads). (C) TEM of cell body of new differentiated exopinacocytes. (D) Semi-thin section of regenerated ectosome with cortical layer and inhalant canals. (E) Semi-thin section of regenerated ectosome and choanosome. (F) Connections of choanocytes of new-formed chamber by interdigitations in their basal parts (arrowheads); (G) TEM of newly-formed choanocyte chamber. (H) TEM of intact choanocyte chamber of the same sponge. Scale bars: A—10 µm; B, C—5 µm; D—20 µm; E—100 µm; F—5 µm; G, H—10 µm. c, cortical layer; cc, choanocyte chamber; ch, choanocyte; exp, exopinacocytes; ic, inhalant canal; iex, intact exopinacocytes; nex, new exopinacocytes.

At 48 h after injury, many newly differentiated exopinacocytes are present at the wound surface, but a continuous exopinacoderm has not yet formed (Fig. 7A). Numerous small cytoplasm microvilli develop at the apical surface of the newly formed exopinacocytes, showing that these cells are motile (Fig. 7B). Cell bodies of these differentiated exopinacocytes are concentrated in the middle layer of the ectosome (Figs. 7C and 7D).

During regeneration of the choanosome, the aquiferous canals and choanocyte chambers form by association of previously disaggregated endopinacocytes and choanocytes, respectively (Figs. 7D and 7E). Cells of each type contact each other and connect by interdigitations (Fig. 7F). Individual choanocytes form groups of cells, forming structures less compact than in the intact choanocyte chamber (Figs. 7G and 7H). Some mesohyl cells still include phagosomes.

**Figure 8:**
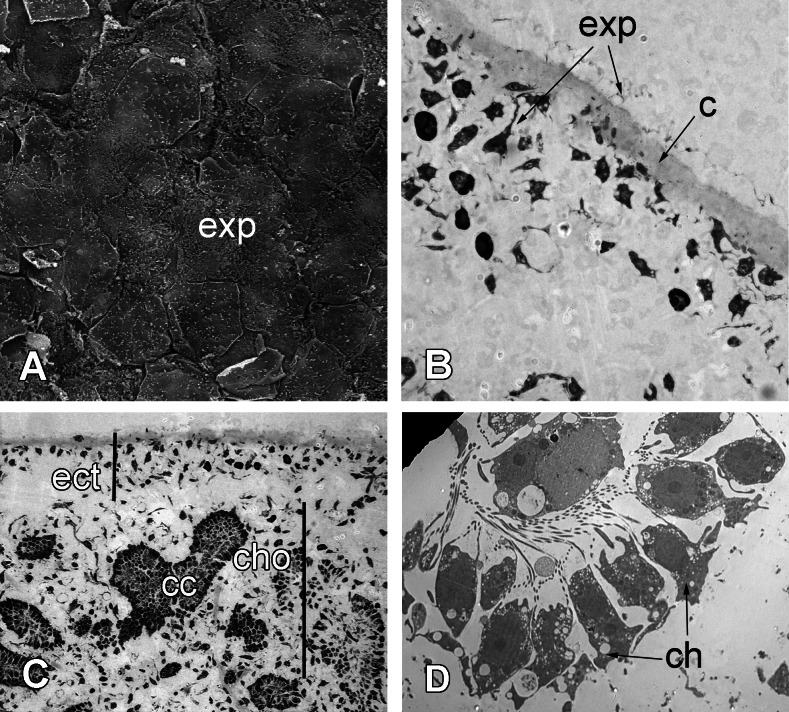
72 h after injury. (A) SEM of the new exopinacoderm. (B) TEM of regenerated ectosome. (C) Semi-thin section of regenerated ectosome (ect) and choanosome (cho). (D) TEM of new choanocyte chamber. Scale bars: A—20 µm; B—20 µm; C—50 µm; D—5 µm. c, cortex layer; cc, choanocyte chamber; ch, choanocytes; cho, choanosome; ect, ectosome; exp, sirface of new exopinacocytes.

Scar-free wound healing is complete approximately 72 h after the injury (Fig. 8). At this point, the new exopinacoderm is identical to the intact one (Figs. 8A and 8B). External flat cytoplasmic parts of exopinacocytes connect with each other by interdigitations. Their apical surfaces are covered with a glycocalyx. All mesohyl cells, as well as the choanocytes and endopinacocytes, are free of the phagosomes and have morphology typical of the intact area (Fig. 8D).

### Cell proliferation during regeneration

**Figure 9:**
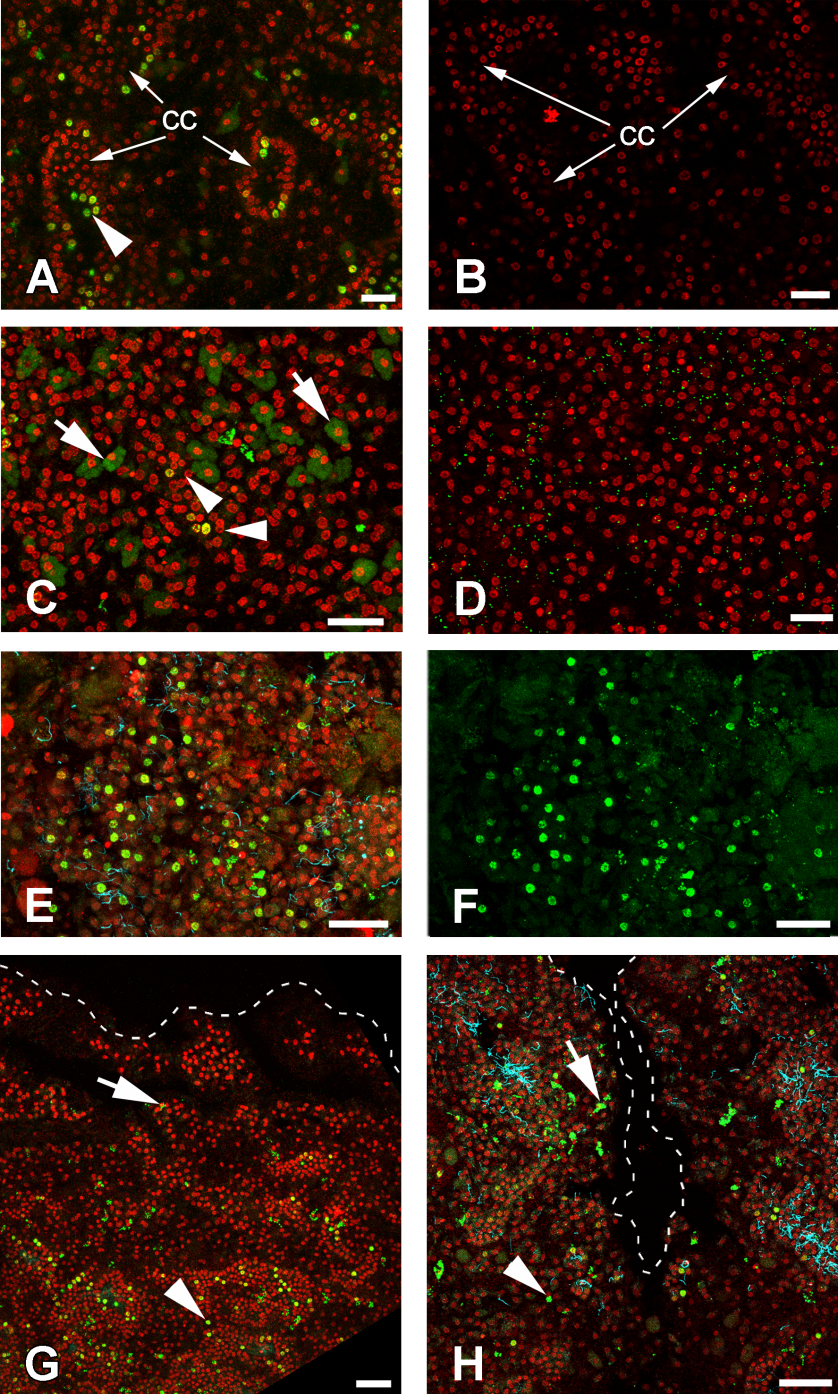
DNA synthesis in unwounded *Halisarca dujardini* and during regeneration. (A) DNA synthesis in choanocytes of unwounded sponge after 6 h incubation with EdU. (B) Negative control for A sample, incubated 6 h without EdU. (C) EdU incorporation in nuclei (arrowhead) and cytoplasm of cells after 24 h incubation. (D) Negative control for C sample, incubated 24 h without EdU. (E), (F) Wound surface after 12 h of regeneration. (F) Green channel (EdU) only. (G) Transversal section of wound surface at 24 h of regeneration. (H) Sagittal section of wound surface at 24 h of regeneration at peripheral level. Wound border outlined with dashed line. Cyan—tubulin, Red—DNA, green—EdU. cc, choanocyte chamber. Arrowheads indicate labeled nuclei, arrows—labeled cytoplasmic granules of unknown nature. Scale bars: A–D—10 µm; E, F—20 µm; G, H—30 µm.

Cells of intact sponges actively incorporate EdU, and after 6 h, labeling nuclei of numerous cells—mainly choanocytes—become marked (Figs. 9A and 9B). Interestingly, long exposure to EdU (20 h and more) results in labeling of the cytoplasm of granular cells, while nuclei of these cells remain unlabeled (Figs. 9C and 9D). This phenomenon has been observed in all sponge samples submitted to long term exposure to EdU (e.g., Fig. 9C).

Many of the choanocytes migrating towards the wound surface after 6 h of regeneration have labeled nuclei (Figs. 9C, 9E and 9F). However, at 12 h after wounding, the labeled cells are absent at the wound surface and underlying mesohyl, although many choanocytes in the deeper chambers are labeled (Fig. 9G). This suggests that choanocytes migrating towards the wound surface to form new exopinacoderm stop their DNA synthesis, while choanocytes in the deeper chambers (even though disorganized) continue to synthesize DNA.

During the reorganization of the aquiferous system between 24 and 48 h following wounding, labeled choanocytes and mesohyl cells are randomly distributed around the wound (Fig. 9H). Thus, at this stage no local proliferation at the wound surface or in deeper layers of mesohyl can be detected. Some choanocytes with their remaining flagella can be found between the newly differentiated exopinacocytes at the wound surface (Fig. 9H).

## Discussion

In this work we show that in *Halisarca dujardini* regeneration follows a sequence of events previously reported for other demosponges (Korotkova & Nikitin, 1969a; Korotkova & Nikitin, 1969b; Thiney, 1972; Diaz, 1979; Boury-Esnault, 1976): (1) closure of injury and disintegration of the structures in the adjacent areas; (2) undifferentiated cell mass (blastema) formation; (3) epithelialization of the wound surface; (4) the reorganization of the inner damaged structures.

The starting point of eumetazoan regeneration is the healing of the wound, which often involves migration of epithelial cells toward the site of injury (Carlson, 2007; Brockes, Kumar & Velloso, 2001).

According to earlier observations in *Halisarca dujardini*, partial epithelialization of the wound takes place within the first 24 h of regeneration, and is completed within 3 days (Korotkova & Movchan, 1973; Korotkova, Suchodolskaya & Krasukevitch, 1983). It has been proposed that epithelization of the wound surface in this species is due to the gradual stretching of the intact exopinacoderm (Sukhodolskaya & Krasyukevich, 1984).

**Figure 10:**
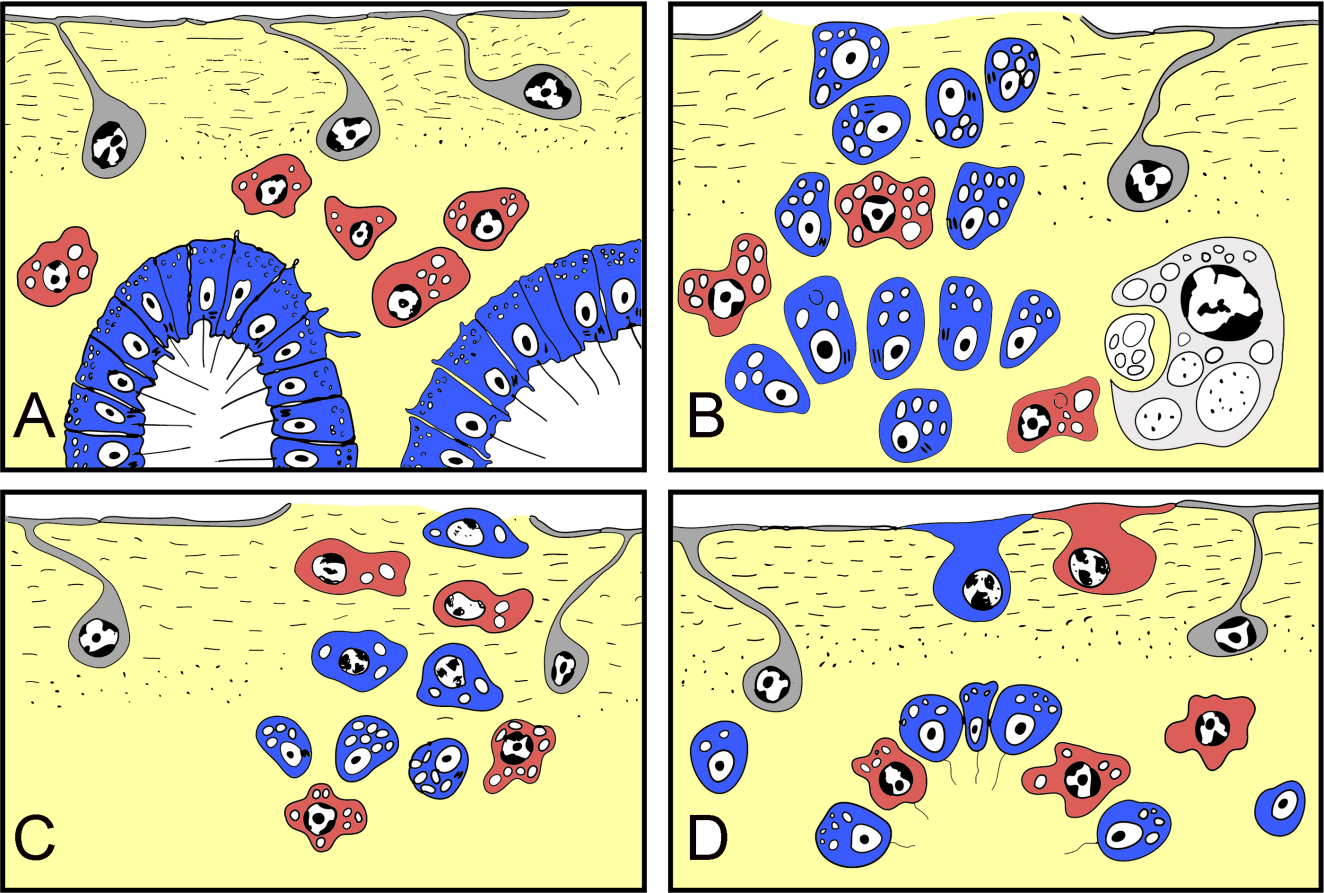
Schematic representation of *Halisarca dujardini* regeneration and the origin of new exopinacocytes and choanocytes. (A) Intact sponge. (B) I stage of regeneration: formation of “regenerative plug”. (C) II stage of regeneration: wound healing and formation of a “blastema”. (D) III stage of regeneration: restoration of ectosome and choanosome. Grey—exopinacocytes, blue—choanocytes, red—archaeocytes.

However, we have demonstrated here that wound epithelialization is due to incorporation of dedifferentiated mesohylar cells and choanocytes, while the peripheral exopinacoderm remains intact (Fig. 10). Until two days after injury, newly differentiated exopinacocytes, covering the wound surface, are motile, as shown by presence of multiple microvilli on their surface.

In regenerative biology, a blastema is defined as a temporary, specialized structure that forms upon amputation or injury and consists of a mass of undifferentiated cells, and which has the capability to form the missing structures by subsequent cell differentiation (Carlson, 2007). In vertebrate limb regeneration, a blastema forms by dedifferentiation of cells of the remaining tissues, which subsequently proliferate, and eventually redifferentiate into the original cell types or transdifferentiate into different cell types (Brockes, Kumar & Velloso, 2001; Vervoort, 2011).

In this work, we refer to the undifferentiated cell mass beneath a wound in *H. dujardini* as a blastema. This temporary structure includes dedifferentiated cells, like choanocytes, endo- and exopinacocytes, and also pluripotent cells—archaeocytes. These cells migrate from the blastema towards the wound surface to differentiate into the new epithelial structure, the exopinacoderm. In the following steps, blastemal cells also form aquiferous system elements. Similar structures have also been described during regeneration of some other demosponges, such as *Halichondria panicea* (Korotkova & Nikitin, 1969a), *Hippospongia communis* (Thiney, 1972) and *Polymastia mamillaris* (Boury-Esnault, 1976).

We found that choanocytes are the most actively DNA synthesizing cells in *Halisarca dujardini* with a few labeled cells (perhaps archaeocytes) also detected within its mesohyl. These findings are consistent with the data available for other demosponges. Study of the choanocyte chamber formation in the freshwater sponge *Ephydatia fluviatilis* showed that choanocytes were rapidly dividing (Tanaka & Watanabe, 1984). De Goeij and colleagues (2009) also showed that in many tropical sponges choanocytes are the most actively proliferating cell population (Alexander et al., 2014). Additionally, in *Hymeniacidon perleve* a high level of BrdU incorporation was observed in isolated archeocytes (Sun et al., 2007).

Importantly, we have not observed increased proliferation of the blastemal cells during *Halisarca* regeneration. These results are consistent with the study of Alexander et al. (2015) on the tropical species *Halisarca caerulea*, where they observed a decrease in the percentage of proliferative cells during early regeneration due to reduction of choanocyte growth phase while the length of the cell cycle remained the same (Alexander et al., 2015). In addition, we show in *H. dujardini* the undifferentiated cells migrating towards the wound surface to form exopinacoderm stop or slow down their cell cycle, while choanocytes in underlying tissues continue to proliferate.

The sources of cells participating in regeneration remain a central issue in research on regeneration (Tanaka & Reddien, 2011; King & Newmark, 2012; Rink, 2013). New cells can be generated in a variety of ways, including (1) proliferation of a resident stem cell population (Weissman, Anderson & Gage, 2001), (2) division of terminally differentiated cells (King & Newmark, 2012), (3) dedifferentiation of mature cells that acts a progenitor cell precursors or other cell types (Jopling, Boue & Izpisua Belmonte, 2011), or transdifferentiation when new cell types arise as a result of a change in state from one cell type into another (Jopling, Boue & Izpisua Belmonte, 2011; Slack & Tosh, 2001). The extent to which each mode is used varies between species and even across tissues within the same species (King & Newmark, 2012).

Traditionally archaeocytes were considered to be toti/multipotent stem cells in demosponges (Buscema, de Sutter & Van de Vyver, 1980; Sun et al., 2007; Funayama, 2013). Archaeocytes were suggested to be totipotent cells by Wilson (1907), Wilson (1910), Müller (1911) and Huxley (1921). Further investigations, mainly at the light microscopic level, supported the idea that archaeocytes are one of the more active actors of exopinacoderm regeneration (Fauré-Fremiet, 1932; Ganguly, 1960; Borojevic & Lévi, 1964; Korotkova & Nikitin, 1969a; Korotkova & Nikitin, 1969b; Thiney, 1972; Korotkova, Suchodolskaya & Krasukevitch, 1983). During the regeneration of *Polymastia mamillaris* incurrent papillae, it was also assumed that the new pinacocytes were derived from archaeocytes (Boury-Esnault, 1976). However, the differentiation of archaeocytes into choanocytes during regeneration has been rarely reported (Brien & Meewis, 1938; Meewis, 1938; Borojevic, 1966). Nonetheless, based on the specific expression of *EfPiwiA* in archaeocytes and choanocytes, it was proposed that both archaeocytes and choanocytes are components of the demosponge stem cell system (Funayama et al., 2010; Funayama, 2013). Under specific circumstances choanocytes transform into archaeocytes, indicating that even when they are fully differentiated, choanocytes maintain pluripotent stem cell-like potential (Funayama et al., 2010).

Here we show that in *Halisarca dujardini* there are three main sources of the new exopinacoderm during regeneration: choanocytes, archaeocytes and (rarely) endopinacocytes (Fig. 10).

One of the unexpected and striking results of our work is that the choanocytes, as well as the archaeocytes, are the main source of new tissue during ectosome regeneration. While archaeocytes directly differentiate into new cells, choanocytes and pinacocytes undergo transdifferentiation during regeneration. This is in line with cell transdifferentiation previously described in *H. dujardini* during somatic embryogenesis (Volkova & Zolotoreva, 1981).

There are two main types of metazoan morphogenesis: epithelial and mesenchymal, involving movement of cell sheets and individual cells, respectively (Gilbert, 2013). Transitions between epithelial and mesenchymal tissue states are referred to as epithelial-mesenchymal transition (EMT) and mesenchymal-epithelial transition (MET) (Hay, 2005; Kalluri & Weinberg, 2009). Both processes are extensively studied in Eumetazoan models, and are involved in embryonic development, asexual reproduction and regeneration (Keller, Davidson & Shook, 2003; Hay, 2005; Radisky, 2005; Kalluri & Weinberg, 2009; Ereskovsky, Renard & Borchiellini, 2013).

Here we show that EMT and MET occur during *Halisarca* regeneration. EMT is the principal mechanism, prominent during the first stages of regeneration (blastema formation), soon after the injury. Epithelial cells (choanocytes and endopinacocytes) from damaged and adjacent intact choanocyte chambers and aquiferous canals assume mesenchymal phenotypes and migrate into the mesohyl. After the blastema is formed, MET becomes the principal mechanism utilized during regeneration of the ectosome and the upper part of choanosome.

Our study demonstrated that regeneration in demosponges involves a variety of processes utilized during regeneration in other animals (e.g., cell migration, dedifferentiation, blastemal formation) and points to particular importance of transdifferentiation in this process. Further studies will be needed to uncover molecular mechanisms governing these processes.

## Additional Information and Declarations

### Data Availability

The following information was supplied regarding the deposition of related data: All data associated with this study are contained in the article.
